# Perceptions and Experiences of Integrated Service Delivery Among Women Living with HIV Attending Reproductive Health Services in Kenya: A Mixed Methods Study

**DOI:** 10.1007/s10461-016-1373-2

**Published:** 2016-04-12

**Authors:** M. Colombini, S. H. Mayhew, R. Mutemwa, J. Kivunaga, C. Ndwiga, The Integra Team

**Affiliations:** 1 Department of Global Health and Development, London School of Hygiene and Tropical Medicine, London, UK; 2 Health Systems Strengthening & Primary Health Care, Centre for Infectious Disease Research Zambia (CIDRZ), Lusaka, Zambia; 3 Population Council, Nairobi, Kenya; 4 Reproductive Health Program, Population Council, Nairobi, Kenya

**Keywords:** Clients’ experiences, Perceptions, Integration, HIV, Kenya

## Abstract

This is one of the few studies that explores preferences of and experiences with integrated sexual and reproductive health (SRH)-HIV care among users of mainstream family planning and postnatal care services who are women living with HIV (WLWH). This paper reports on the quantitative data from 179 clients attending public sector clinics and from 30 qualitative in-depth interviews with WLHIV in Kenya. Quantitative data show that integration is happening for the vast majority of these clients at their last HIV visit. However, qualitative data show that very often the care received by WLWH is fragmented as providers do not offer multiple same-day appointments for FP and ARV refills. Our study has shown factors that could either prevent or enable receipt of integrated SRH and HIV care for WLWH. To address these factors, management systems need to be able to support providers to make flexible decisions and facilitate better coordination and communication across clinics within facilities.

## Introduction

With the new treatment guidelines recommending earlier ART initiation and all pregnant and breastfeeding women to start lifelong ART [1], millions of WLWH begin or continue to get ARVs for their entire reproductive lives [2]. Moreover, evidence shows an unmet need for family planning (FP) and unwanted pregnancies among WLWH [3–6]. Therefore, it is important that their sexual and reproductive health (SRH) needs are fully addressed through integrated SRH and HIV services.

Many studies and reviews have assessed the effectiveness of the integration of HIV and SRH services, discussing issues around coverage, service uptake and time-to-treatment initiation [7–14], access to quality services [15], providers’ experiences and willingness to deliver joint services [16–23], cost-effectiveness [24, 25], and health systems barriers to integration [2, 3, 26, 27].

Some have also reported patients’ acceptability, preference of and satisfaction with integrated care [28–33]. For example, findings from Kenya indicate that integration of ANC and HIV services is highly desired by WLWH as it improved their overall clinical experience [28]. Similarly, a qualitative study from South Africa on clients’ perspectives on integration of HIV and SRH care suggests a preference for integrated care among female clients, particularly because of stigma reduction and higher access to contraception [29]. Some of the positive gains of integrated care reported by women include increased coverage of HIV testing, improved convenience, efficiency, confidentiality, and increased likelihood of using a family planning method, if provided at an HIV clinic [31, 34]. However, the evidence on the clear impact of integrated services on patients’ satisfaction and stigma is mixed [15, 35, 36].

Less information exists on patients’ experiences with integrated care, primarily among pregnant women [26, 34, 37]. A study on improving the linkage of HIV-positive pregnant women to long-term HIV care and treatment in eastern Uganda reports that facilitating factors for linked care include (among others) support from expert clients, escorted referrals, and coordination between ANC and HIV service; while reported barriers for women included shortages in HIV testing kits and fear of social, physical and medical consequences [26].

This study therefore aimed to address this evidence gap on patients’ actual experiences with integration and analyse HIV positive female clients’ attitudes to and experiences of integrated SRH-HIV care.

## Methods

### Study Site

Kenya suffers from a generalised HIV epidemic affecting women disproportionately: 6.9 % of women compared with 4.2 % of men [38]. Kenya is one of the few countries in Sub-Saharan Africa that adopted a national policy framework on HIV/SRH Integration [39, 40].

### Methodology

The study formed a subcomponent of a multi-country study investigating the benefits and costs of service integration in Sub-Saharan Africa (Kenya and Swaziland), the Integra Initiative [41]. The parent study conducted four rounds of data collection with these clients (and two rounds of qualitative interviews). This paper reports on the data from clients attending sixteen public sector clinics in Kenya. The data reported here are from the last round of survey questionnaires and the last set of linked in-depth interviews (IDIs). Both of these took place approximately 24 months after recruitment—this means that the “PNC” cohort were no longer PNC clients but had progressed to become FP clients or non-clients. Therefore, the label “SRH” is going to be used instead for all the interviewed clients, whether attending FP or PNC services.

### Definition of Integrated Care

Integrated care was defined as receipt of an HIV care service (counselling, testing, and antiretroviral therapy (ART), and CD4 count) AND a contraceptive method (condoms, short or long-terms methods) in the same visit.

### Quantitative Data

#### Data Collection

A closed-ended questionnaire was used to collect data on women’s fertility intentions, pregnancy, use of FP, other SRH and STI/HIV-related behaviors and health-seeking behaviors. Women were also asked whether they ever had an HIV test, whether they knew their status, and if so, whether they were willing to voluntarily disclose their status. There was no pressure for them to disclose their HIV status and unwillingness to do so was not a criterion for exclusion from participating in the INTEGRA study. Trained research assistants conducted the interviews using hand-held personal digital assistants (PDAs) loaded with the questionnaire tool translated into Swahili. Every respondent was given a full description of the study and gave their informed consent in writing prior to interview.

Apart from the recruitment interview, which was held at the health facility, follow-up interviews were conducted outside the facilities, unless explicitly requested by the interviewee, to minimise service-related courtesy bias. Written informed consent (or in a few cases witnessed thumb-printed consent) was obtained from all participants before they were interviewed, each time they were interviewed.

Due to missing data on 61 women, the quantitative analysis covered 179 of the 240 WLHIV in the cohort. Given the descriptive nature of the analysis, we did not think the results are significantly affected by the dropping of cases with missing data. Also many of the cases had data missing on several key variables and thus imputation was considered inappropriate. 240 WLHIV were interviewed in Kiswahili between March and July 2012. Many of the clinics were already integrating services and thus we did not separate clinics by level of integration.

#### Quantitative Analysis

For this analysis, only HIV-positive disclosed women were included (n = 179), irrespective of whether they attended postnatal care or family planning services. A table describing key socio-demographics characteristics of the sample by service attended was generated. The combined sample was subsequently descriptively assessed for: receipt of integrated care at last clinic visit, type of family planning method received during the last visit, and overall satisfaction with services.

In order to assess distributional differences on key service satisfaction indicators between women who received integrated care and those that did not, a separate table was constructed for each of the two sub-populations. All tables are descriptive, no statistical analyses for significance were performed due to the small numbers involved over the nine service satisfaction indicators used and each measured across the three levels of the Likert scale deployed. The small counts could only guarantee inefficient inferential results, hence the use of descriptive statistics. The descriptive statistics were obtained from the data using Stata Version 13.1 [42].

#### Qualitative Data

Thirty qualitative IDIs were conducted with women living with HIV (WLHIV) who were sampled from the quantitative cohort described above. Qualitative respondents were purposively selected to ensure representation from a wide range of study facilities [15]. In total 13 respondents were interviewed from 7 of the possible 12 study facilities in Central Province and 17 respondents were interviewed from 9 of the possible 12 study facilities in Eastern Province. 18 selected HIV-positive respondents from the original qualitative cohort could not be interviewed in this second round because some respondents declined consent (due to fatigue), could not be traced (relocated from the residence at the time of interview) or had died. It was not possible to recruit women from every study facility because we did not have self-disclosed HIV positive women at every site.

A topic guide was developed based on the literature on experiences and practices of integration among WLHIV, some preliminary analysis of the round 2 quantitative survey data and the core Integra objectives. The guide explored respondents’ views on integrated care; views and experiences at last SRH visit, and challenges around integrated care. This guide was pre-tested in early 2012 among 8 respondents at one (non-study) clinic. Subsequently it was refined and 8 local interviewers were given a 4-day training (by Integra research partners) on qualitative data collection, the topic guide and interview practice and critique.

Face-to-face interviews were then conducted in Kiswahili in February 2012. The selected respondents (from the quantitative survey) were asked to reconfirm their consent for an IDI. Interviews were conducted in private locations chosen by the interviewees. Most took place in the respondents’ homes; none took place in health facilities (minimising the risk of service-related courtesy bias). The interviews were designed to take approximately 1 h to complete. All interviews were audio-recorded, transcribed then translated into English. The transcripts were coded by the authors using NVivo 9.0 and analysed using thematic analysis which was exploratory and inductive (not deduced from a pre-existing theory).

### Ethical Clearance

Ethical clearance was granted by the Kenya Medical Research Institute (KEMRI) Ethical Review Board (#113 and 114), the Ethics Review Committee of the London School of Hygiene & Tropical Medicine (LSHTM) (#5426) and the Population Council’s Institutional Review Board (#443 and 444). The Integra Initiative is a registered trial: ClinicalTrials.gov Identifier: NCT01694862.

## Results

### The Quantitative Sample

Table 1 presents key characteristics of the survey sample, which was composed of 179 women. The majority of the clients were aged 25 and above (95 %); married (64 %) and employed (72 %); the largest group was on ART for over 24 months (72 %); the great majority not wanting any more children after discovering their positive status (73 %).

**Table 1:** Socio-demographic characteristics of HIV positive survey sample (n = 179)

Factor	n (%)
Type of client	
HIV/FP	151 (84)
HIV/PNC	28 (16)
Age	
16–25	9 (5)
26–35	89 (50)
36+	81 (45)
Marital status	
Single	64 (36)
Married	115 (64)
Education	
Up to primary	126 (70)
Above primary	53 (30)
Employment	
Unemployed	50 (28)
Regular	116 (65)
Professional	13 (7)
Household income	
<KSh3000	59 (33)
3000–9999	92 (51)
10000+	28 (16)
Currently on ART	
No	38 (21)
Yes	141 (79)
Months since starting ART	
Up to 12 months	27 (19)
12–24 months	13 (9)
Over 24 months	101 (72)
Knowledge of partner’s HIV status	
No	42 (25)
Yes	129 (75)
Number of living children	
<3 children	88 (49)
3 children	48 (27)
>3 children	43 (24)
Desire for children after testing HIV+	
Never have a child again	83 (73)
Have a child after careful planning	28 (25)
Quickly have another child	2 (2)
Received integrated care in same visit	
No	61 (34)
Yes	118 (66)

Qualitative sample was drawn from this sample

### Receipt of Integrated Service-Delivery

The vast majority of the clients received integrated care at their last HIV visit (66 %). Table 2 illustrates that the largest group (56 %) who received integrated care (FP method at their last HIV visit) received one of the following short-term contraception methods: hormonal pills, injectables, emergency contraception or a natural family planning method. It was also found that over half of the clients (61 %) were not recommended any FP method by their providers, and very few were recommended either short (9 %) or long-term contraception (9 %). No difference appeared to exist in receipt of long-term methods between the clients who got integrated care versus the ones who did not.

**Table 2:** Proportion of women who received integrated care at last visit, by type of FP method

FP method	FP method provider recommended	FP method received (integrated service) n (%)	FP method received (not integrated service) n (%)
No FP method used	–	–	6 (10)
No FP method recommended	110 (61)	–	–
Condoms	36 (20)	32 (27)	20 (33)
Short-term	17 (9)	66 (56)	25 (41)
Long-term	16 (9)	20 (17)	10 (16)
Total	179	118 (66)	61 (34)

Integrated service: receipt of an HIV care service (counselling, testing, and antiretroviral therapy) and a FP method; condoms = male condoms, female condoms; short-term methods = hormonal pills, injectables, foaming tablets, emergency contraception, natural family planning; long-term methods = IUCD, implants, sterilization, diaphragm/cap

### Satisfaction with Services Received

Tables 3 and 4 display clients’ overall satisfaction with the services received at the clinic they regularly attend over time and they show that satisfaction is high among all clients. In both sets of clients—irrespective of receipt of integrated care—more than two-thirds think they are able to receive more than one health care service from the same provider during a visit. Overall, clients show mixed feelings about the existence of privacy in the waiting room, and about separation of HIV services from other services. However, women who receive integrated care have slightly lower fear of involuntary disclosure at their clinic (19 %) than the ones who did not receive integrated care (27 %).

**Table 3:** Satisfaction with services among women who received integrated care

N = 118	Agree, n (%)	Disagree, n (%)	Mixed feelings, n (%)
I’m happy overall with the services here	108 (92)	4 (3)	6 (5)
Waiting times are long	40 (34)	70 (59)	8 (8)
I am able to receive more than one health care service from the same provider (in a visit)	79 (67)	28 (24)	11 (9)
Health workers in this facility cannot be trusted to keep my records confidential	19 (16)	84 (71)	15 (13)
My consultation was not private	25 (21)	90 (76)	3 (3)
Doctors/nursing staff are not always available	16 (14)	88 (75)	13 (11)
Others can find out my status when I come to this clinic for HIV services	23 (19)	82 (69)	13 (11)
It bothers me if other people in the waiting room know my status	51 (43)	55 (47)	12 (10)
It is better if HIV services are separated from other health services	55 (47)	59 (50)	4 (3)

**Table 4:** Satisfaction with services among women who did not receive integrated care

N = 61	Agree, n (%)	Disagree, n (%)	Mixed feelings, n (%)
I’m happy overall with the services here	50 (82)	3 (5)	8 (13)
Waiting times are long	17 (29)	36 (59)	8 (13)
I am able to receive more than one health care service from the same provider (in a visit)	45 (74)	6 (10)	10 (16)
Health workers in this facility cannot be trusted to keep my records confidential	12 (20)	43 (70)	6 (10)
My consultation was not private	11 (18)	48 (79)	2 (3)
Doctors/nursing staff are not always available	10 (16)	43 (70)	8 (13)
Others can find out my status when I come to this clinic for HIV services	16 (27)	38 (63)	6 (10)
It bothers me if other people in the waiting room know my status	24 (40)	26 (43)	10 (17)
It is better if HIV services are separated from other health services	28 (47)	29 (48)	3 (5)

### Exploring Integrated Care and Clients’ Preferences

While the quantitative data demonstrate that a large amount of SRH-HIV integration is happening and there is high satisfaction with integrated care among clients, other aspects seem to affect the actual reception of integrated services and client’s experiences with it. Qualitative data helped us explain the breadth of reasons behind receipt or non-receipt of integrated care by WLWH and elucidated clients’ experiences of and preferences for integration. Thirty women were interviewed qualitatively, the majority of which were married or with a partner (77 %). Over half of them (57 %) had 3 or more children. The qualitative subsample was not significantly different than the larger sample presented in Table 1.

### Appreciation of Integrated SRH-HIV Care

Many WLWH reported appreciation of receiving SRH and HIV services together. When asked if clients preferred having all services received during last visit together or separately on different days, many mentioned they would rather have all services on the same day. Many conceived ‘integration’ as receiving all services ‘on the same day’ at the same facility, but not necessarily by the same provider. The most prominent reasons for preferring integrated care were to save time and money (to save bus fare and reduce time off at work).

“If I was to be asked, I would prefer, even the Norplant should be done here [CCC^1^] because when you come in the morning… a lot of people come in the morning. […] you see you must come very early in the morning. Because if you do not come early, you will get late to queue in all other places” [010464, 2 children, married, on ARVs].

“[yes] I would like to finish everything at one go and not to come back. […] You see I came on 22nd and the child came on 23rd… The problem is I am coming from far and I am using fare and being told come today, tomorrow I use a lot of money. And at times I end up not coming because of lack of fare” [140448, 3 children, married, on ARVs].

“Let’s say today I am going for family planning and I am using vehicles… That is money. Tomorrow family planning, the following day for HIV virus, that way it becomes expensive” [081125, 3 children, married, injection, on ARVs].

### Long Queues at Family Planning Clinics Discouraged Clients’ Preference for Integration

Perception of long waiting time due to integrated services (primarily due to FP services) was mentioned by a small minority stating they would prefer to have return visit than waiting for FP services as there would be long queues. Two clients said separation of services could help reduce waiting time (when talking about tube ligation and child services): services have their ‘own days’ and are not mixed up otherwise ‘one *would stay there till night’*. This is in contrast with the majority of other clients saying that integration saves time and reduces waiting time.

#### Elements that Contributed to Actual Receipt of Integrated Care

Nearly half of WLWH interviewed reported receiving integrated services (defined as ‘same-day services’) during their last visit. The majority of these were using short term FP methods (including condoms, pills and injection) and went to the clinic to obtain HIV care and/or FP services including FP refill of short-term methods, and ARV refills. Only four clients reported using either long-term or irreversible FP methods (implant and tube ligation) or no FP method, and thus only received HIV care at CCC or VCT. As people discussed ‘integrated SRH/HIV care’, various factors emerged that seem to have influenced clients’ receipt of joint services on the same day.

Client’s active demand for additional services and provider’s willingness to offer immediate referral seemed to impact on actual receipt of integrated care. During her last visit to the HIV Unit (Comprehensive Care Centre—CCC) to pick up her ARVs, one client (on ARV for 4 years and currently using norplant) also received family planning services. The key reason for receiving integrated HIV and FP services for this client was that she took the initiative to ask the HIV provider to check her implant and therefore she got referred to the FP clinic for implant check. [040535, on ARV for 4 years, current FP user (norplant), 2 children, separated and single]

Many who reported receiving integrated services at their last visit, also reported that FP and HIV services are usually offered on separate days at their facilities. For many, it seemed that integration of FP and HIV services on same day during the last visit was more due to luck than actual coordination (over appointments) across services and dates for both FP and HIV.

“I: Is there another time you have been served and had to come on another day for a different service?

R: You know you can go today and it is the day for drugs and the day for the [FP] injections has not reached then you will have to come that other week [120258, 2 children, married, on ARVs].

“I: Is the day you go for FP the same as the one you go for HIV services?

R: No it’s not the same.

I: But how about the last visit?

R: They coincided” [10 03 88, 3 children, married, on ARVs].

“R: Because the time [date] I go for HIV/AIDS drugs/services is not time [date] for going to family planning services; there is a separate date for family planning and CCC services […].

I: Have you ever received FP services at CCC?

R: Yes, I get sometimes when you go and say you need services and it’s your date for FP, they give you” [060208, 2 children, in relationship, on ARVs].

The type of FP method used by WLWH also seemed to impact on the integration of FP care within specialised HIV services. In particular, less clinical methods, such as condoms and oral pills, were reported to be available at HIV services, making it possible for some clients to experience functional integration of HIV care (primarily ARV collection) and FP services (primarily FP counselling, condoms and oral contraception). In some instances, women reported that condoms and pills were usually offered at CCC and at the same visit. Others reported condoms and counselling on dual protection were offered at CCC, but by separate providers: HIV ones and FP ones.

### Constraints for Having Integrated Services

Less than half WLWH [11] did not receive integrated SRH/HIV care for various reasons including having received FP and HIV services on separate days during their last visit; or using different facilities for FP and HIV. A small number only needed HIV care as they did not have any FP need (TL or implant or no FP use).

Women who used a different dispensary for FP services cited proximity (thus impacting on costs), quality of care offered and fast services as the main reasons for opting for separate SRH/HIV services.

I like going to [name of dispensary] because if you go there [at the FP private dispensary] they serve you very fast and then you go… because it doesn’t have many people… and because they charge very little. You know at the general hospital [where she goes for HIV care] there are many people so you can be at the hospital until 4.00 pm [0105 45, 3 children, married, on ARV, FP user].

I: Why did you specifically chose to come to [name] district hospital for the HIV care?

R: Because this is where I was tested from when I was sick and began the clinic. […] We don’t have any in our place that offer the HIV care services”.

[…]

I: Why did you just choose [name] dispensary?

R: Because the one that I used to go in before [name], the last time I went for an injection, it was not effective. I still got pregnant. This is why I shifted to a different facility” [070586, 5 children, on ARVs, married, FP user].

### Nature and Timing of Services and Lack of Coordination or Appointments Across Services

Despite clients’ preferences for having all services in one visit, some women reported that the nature of some of the services may not lead to an easy integration, stating that, for instance, it would be problematic to have a single visit to get 3-month injection and collect 2-months ARVs supply.

I get one service after two months and this other one after 3 months. So it may be better to just have two days because, if I get both services in one day, I will need to go for HIV services only, before I go for family planning services since I need HIV services every two months and family planning services every 3 months. Therefore when it is time to go for HIV services, there is still one month to go before I need to go for family planning… because they come separately [010595, 3 children, on ARV, FP user].

The above quote suggests the need for a tailored FP cycle; for instance, collecting 2-month worth of pills every time she comes for ARV refills. However, this integration requires not only coordinating the appointments system between HIV and FP services, but also changing the usual FP dispensing regime requiring a degree of initiative from the provider’s side.

In another instance, integration seems to be achieved only providing that every third month the injectable is given at the same time as the visit for ARV refill. In this case, it is the ARV regimen that needs changing (to 3 monthly rather than 2 monthly). The client reported getting FP every 3 months and ARVs every month; thus her last visit was only for ARVs, while in the previous one she went for FP injection and also got ARVs [140448, married, on ARVs, FP use, 3 children].

Some women who reported their preference for integrated services also recognized that it could be difficult to have integration because of the system of appointments in two different clinics and the differences in service dispensation (pick-up routines for refill) between ARVs and FPs. This shows the importance of discussing not only what type of FP method is suitable, but also its timing.

I: Yes, in one day you receive all the services you want in [name of facility]… would you like that?

R: eeh… [Yes] that can be very good indeed. Because you know… let’s say today you have come to take ARVs then tomorrow you will go for family planning… you know that becomes costly and as you well know bus fare is always a problem.

I: Okay and which services would you like to receive at the same time?

R: May be I can talk to the health provider so that when they give me an appointment for ARV refill they also give me an appointment for family planning services. But usually they give you an appointment for a particular date every month. If you pick your medicine on the fourth then all the health providers who give you the different services can give you appointment on the fourth of every month so that you will always be coming for medication for all your health needs on that day. But that would not be possible because the ARVs must be picked every month and family planning services may not be picked every month [07/05/45, married, on ARV, FP use, 2 children].

Lack of awareness about the possibility of receiving integrated SRH/HIV services

Some women did not seem to be aware of the possibility of receiving integrated services and thus they did not have any need to demand for more services together.

FP is what had taken me and this is what I got. [….] I had gone there only for FP and my problem was FP, so I could not ask anything more than FP [01/02/114, 1 child, not on ARV].

There seems to be no promotion by health providers about integrated services and clients may not feel they could ask for additional services. Only two clients stated they openly asked their providers to get an appointment for both FP and ARV collection on the same day [140433 and 070545].

## Discussion: Implications for Delivery of Integrated Care

This is one of the few studies that explores preferences of and experiences with integrated SRH-HIV care among users of mainstream FP and PNC services who are WLWH.

Quantitative data show that integration (i.e. receipt of multiple services in one visit) is happening for the vast majority of these clients at their last HIV visit, and more than half of the clients believe they can receive integrated services. The fact that these are WLWH who have multiple needs may mean they are particularly aware of the possibilities for multiple service-access. Overall, clients who received integrated care show slightly higher satisfaction of services though it is high for all WLWH. The quantitative data, however, shows mixed feelings about clients’ preferences for separation of HIV services from any other services with responses being split almost 50:50 for and against. Although clients did not explicitly identify integration as being important in their judgment of the services, there were clear indications from many of their qualitative responses that they would appreciate a more integrated approach in their SRH and HIV services. Their preference for joint care was primarily due to time and financial savings. This reflects other Sub-Saharan African studies on integration of care indicating clients satisfaction with receiving a broader array of services under one roof [15, 28, 29]. Another study, however, nested within the main Integra study, found that in Swaziland satisfaction was highest at a fully stand-alone HIV-clinic indicating that HIV clients appreciate health services in different ways, depending on their needs and situation [32]. Our study in Kenya seems to show greater acceptance of integration as a good model of care for WLWH, with women being less worried about privacy and confidentiality, and interpersonal care, and instead more focused on reducing time and financial constraints of repeated appointments. Nevertheless, almost half wanted HIV services separated from mainstream services, suggesting greater integration of FP/RH services *within* HIV-specialist services, as well as those available in mainstream facilities/units would provide greater client choice.

### Synchronising Care for Joint Appointments

It transpires from the findings that integrated care does not always happen automatically, even when services are supposed to be integrated. Several clients interviewed experienced fragmented care where SRH and HIV visits were not joint. Systems level issues comprise time spent queuing, no joint appointments system due to lack of coordination across clinics/units so dates for both FP and HIV often do not coincide. Our findings show that patients’ constraints in having integrated SRH-HIV care are mostly affected by lack of coordination and communication among different health facility units/departments (e.g. FP and HIV), due to organisational issues that impede facilities’ ability to provide joint SRH/HIV appointments. Integration also seems to be affected by the way clinics are often organised having specific ‘clinic days’ for each service (e.g. FP days, ANC days), also showed elsewhere [32].

**Fig. 1:**
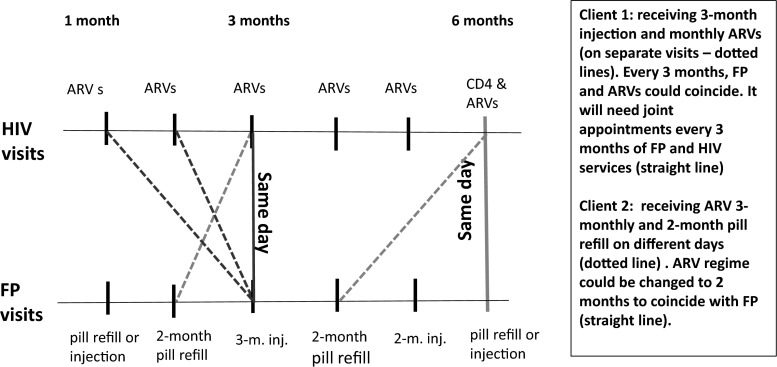
Implications for integrating FP short-term methods and HIV services (ARV refills)

In general, it seems that complications with integration services arise when trying to combine ARV collection (most often offered monthly or in few cases 3-monthly) with short–term FP methods like the pill or injection, usually offered every 2 or 3 months. Our findings, to some extent, show a tension between the timing (when one actually needs these different services) and the appointment and collection system for each regime. Figure 1 shows the practical implications for trying to integrate FP and ARV regimes. The synchronisation of start dates for FP methods and ARVs is critical: if ARVs are monthly then every 3rd visit would be an injectable as well, but if the injectable was started half way through the month they will never coincide unless 1.5 months of ARVs can be provided to reschedule ARV collection dates. Ideally, a synchronised and coordinated pick-up system would need to be created that takes into account the timing of the FP and the ARVs collection. For example, if ARVs are collected monthly it should be possible to coincide most FP methods visits around this (once initial synchronisation has been ensured). If ARVs are dispensed every 2 months, it might be more difficult, but it could work with pills and long-term methods and could also work for every second visit of 3-month injectable.

Collection days and treatment course often vary by clinic and by client (with initial adherence monitoring for ARV clients often requiring more frequent visits then collection dates eventually lengthening) and thus planning joint or coordinated services must be done at facility level on a client by client basis, requiring staff capacities to take initiative to link services across services—something we found virtually no evidence of. In Kenya, some large facilities offer ARV services daily and have large clientele, while some smaller ones—e.g. health centres and dispensaries—offer them on a specific day depending on the numbers of clients and availability of staff (a staff trained on ARVs could be shared between 2 facilities on different days). Clients could get appointments every 2 weeks, monthly and up to three months, depending on the level of ARV initiation (e.g. early stages of ARV or those well settled with the treatment) and how clients are doing on ARVs (personal communication with key informant from Population Council’s Kenya office). Therefore, integrating appointments for joint FP and HIV services may not be straightforward and needs careful planning. Specific standard FP regimens (for the different types of contraceptives) for WLWH on ARVs should be developed to facilitate coordination of appointments and collection. District systems would need to buy into this too as it would affect supply chain regularity/ordering time schedules, etc.

### Challenges and Enabling Factors for Integrated Delivery of Care

At individual level, issues include the fact that providers do not promote integration to clients, and only half of clients in this generally aware group, knew of the possibility of receiving integrated services. Lack of time by providers to offer additional services could be a constraint (as stated in a study in Nigeria [43]), together with the type of training received by healthcare staff, which seems very technical around clinical service integration, but failing to focus on basic issues around service offer and promotion of integrated care.

Furthermore, we found clients did not question the system. Cultural norms where acceptance of a top-down approach to authority, allied with deference to those with specialist medical knowledge [27, 44], means that clients may not voice their desires for integrated services and also will not criticise the misgivings they have of a fragmented service. Our qualitative findings showed the majority of our respondents valued the idea of receiving joint FP and ARV/HIV services, but this is not articulated as client requests because of low awareness that integrated services could be provided if requested and a lack of confidence among clients to ask for them or challenge medical staff. A community survey, conducted by Integra, on the need and demands for SRH and HIV services in Kenya and Swaziland showed some “latent demand” among clients for integrated care but high levels of unmet need for FP and HIV prevention and missed opportunities for integration. It highlighted the important role of provider-initiated integrated care to cover a broad range of SRH and HIV services when clients attend [45].

In our study, enabling factors affecting receipt of integrated care include ‘luck’ of having coinciding dates for SRH and HIV services, clients’ active demand for additional services and providers’ willingness to offer them (individual agency and willingness), and the type of method used where less clinical ones (condoms and oral pills) are more likely to be integrated within HIV services.

This highlights the need for additional training for HIV providers to include FP methods that require higher-level clinical skills (e.g. IUDs). If integration is to be extended efficiently, it is crucial to include provision of all types of contraception (clinical versus less clinical ones). However, demand for such services needs to be increased to justify such training. For instance, a study on providers’ mentoring as strategy for capacity building in SRH and HIV integrated care in Kenya found that some providers had difficulty learning less common procedures such as cervical cancer screening, syndromic management of STIs, and IUCD insertions because they did not have many clients requesting these and this hindered their ability to practice the new skills [46].

### Limitations

The study has limitations. Quantitative data are primarily descriptive, however, they help contextualise the qualitative data. Although it is not possible to generalise from the qualitative data presented, these offer important insights that are worth researching in other settings. We have not been able to return to verify whether any of the identified barriers have been removed, though anecdotally from partners on the ground it seems that they have not. Follow up work would thus be useful to identify if and how barriers were overcome. Furthermore, since a self-selected sample of WLWH was used, it is likely that it may not have captured views from all the people who were positive. Moreover, failure to confirm HIV status among study participants is a limitation; however, it is unlikely to have impacted the qualitative data as those participants would have struggled to fake the discussions with interviewers and indeed talked in some detail about their experiences with HIV-related services.

## Conclusions

WLWH who are using FP and HIV services within a mainstream setting appreciated the integration of these services. Our study suggests, however, that very often the care received by WLWH is fragmented as providers do not offer multiple same-day appointments for FP and ARV refills. Our study has explored factors that could either prevent or enable receipt of integrated SRH and HIV care for WLWH. To address these factors, management systems need to be able to support providers to make flexible decisions and facilitate better coordination and communication across clinics within facilities in order to allow, for example, tailored FP and ARV collection regimens that are amenable to joint appointments.

## Footnotes

1 CCC are Comprehensive Care Centres where HIV positive clients receive HIV care and treatment.
